# Investigation of Antibody-Dependent Enhancement (ADE) of SARS coronavirus infection and its role in pathogenesis of SARS

**Published:** 2011-01-10

**Authors:** Ming S Yip, Chung Y Cheung, Ping H Li, Roberto Bruzzone, JS Malik Peiris, Martial Jaume

**Affiliations:** 1 HKU-Pasteur Research Centre, Hong Kong, Hong Kong SAR; 2 Department of Microbiology, The University of Hong Kong, Hong Kong SAR

## 

Antibody-dependent enhancement (ADE) is a mechanism by which viruses, such as dengue, HIV and Ebola, gain entry into some target cells through the use of host antiviral humoral immune responses [1]. Here, we studied the ability of severe acute respiratory syndrome coronavirus (SARS-CoV) [2] to use ADE mechanisms to enhance its infectivity towards cells of the hematopoietic lineage.

We found that heat-inactivated immune serum from rodents vaccinated with recombinant native full-length Spike protein trimers [3] triggered infection of human immune cells (monocytic and B cell lines) by SARS-CoV Spike pseudotyped particle (SARS-CoVpp). The occurrence of antibody-mediated infection of human Raji B cells was further investigated by using live SARS-CoV. Similarly to results obtained with the SARS-CoVpp, only anti-SARS-CoV Spike serum, but not mock immune-serum, induced a massive increase of SARS-CoV viral genes (ORF1b and Nucleocapsid) and viral proteins (Membrane and Nucleocapsid) in Raji B cells. As revealed by immunostaining, only a relatively low, however significant percentage of the Raji cells get infected by antibody-mediated infection and did not allow direct assessment of productive replication by conventional cytopathic assays and TCID50 titration.

Taken together, our data suggested that SARS-CoV is able to enter human immune cells via an antibody-mediated pathway and immunological consequences of such infection are under investigation (productive replication, cytokines secretion profile and cell death etc). Our data raise reasonable concerns regarding the use of SARS-CoV vaccine in humans and pave the way to further studies focusing on the role of immune-mediated infection phenomenon during SARS pathogenesis.
